# High Serum Carotenoids Associated with Lower Risk for Bone Loss and Osteoporosis in Post-Menopausal Japanese Female Subjects: Prospective Cohort Study

**DOI:** 10.1371/journal.pone.0052643

**Published:** 2012-12-20

**Authors:** Minoru Sugiura, Mieko Nakamura, Kazunori Ogawa, Yoshinori Ikoma, Masamichi Yano

**Affiliations:** 1 Citrus Research Division, National Institute of Fruit Tree Science, Shizuoka City, Shizuoka, Japan; 2 Department of Community Health and Preventive Medicine, Hamamatsu University School of Medicine, Hamamatsu City, Shizuoka, Japan; Oklahoma State University, United States of America

## Abstract

**Introduction:**

Recent epidemiological studies show that high intakes of carotenoids might be useful to maintain bone health, but little is known about the association of serum carotenoids with change of bone mineral density (BMD). The objective of this study was to investigate longitudinally whether serum carotenoids are associated with bone loss.

**Methods:**

We conducted a follow-up on 146 male and 99 pre- and 212 post-menopausal female subjects from the Mikkabi study. Those who participated in previous BMD surveys and completed four years of follow-up were examined longitudinally.

**Results:**

During a 4-year follow-up, 15 of the post-menopausal female subjects developed new-onset osteoporosis. In contrast, none of the male and pre-menopausal female subjects did. In male and pre-menopausal female subjects, the six serum carotenoids at the baseline were not associated with bone loss. On the other hand, in post-menopausal female subjects, the 4-year bone loss of radius was inversely associated with the serum carotenoid concentrations, especially in β-carotene. After adjustments for confounders, the odds ratios (OR) for osteoporosis in the highest tertiles of serum β-carotene and β-cryptoxanthin against the lowest tertiles were 0.24 (95% confidence interval 0.05–1.21) and 0.07 (CI: 0.01–0.88), respectively. Serum β-cryptoxanthin was also inversely associated with the risk for osteopenia and/or osteoporosis (*P* for trend, 0.037). In addition, our retrospective analysis revealed that subjects who developed osteoporosis and/or osteopenia during the survey period had significantly lower serum concentrations of β-cryptoxanthin and β-carotene at the baseline than those in the normal group.

**Conclusions:**

Antioxidant carotenoids, especially β-cryptoxanthin and β-carotene, are inversely associated with the change of radial BMD in post-menopausal female subjects.

## Introduction

Osteoporosis and related fractures are a major public health problem [1]. Osteoporosis is a chronic disease characterized by low bone density and microarchitectural disruption, leading to bone fragility and an increased susceptibility to fractures [2]. Nutrition is an important modifiable factor in the development and maintenance of bone health, and numerous studies on nutrition and bone health have been conducted [3], [4]. With regard to nutritional approaches to bone metabolism, calcium and vitamin D have been identified as important nutritional factors to maintain normal bone metabolism. Other nutrients, such as potassium, magnesium, zinc, copper, iron, vitamin C, and vitamin K, may also have beneficial effects. Fruit and vegetables are rich sources of these nutrients for bone metabolism. Therefore, the intake of these types of food might affect bone health. In fact, recent epidemiological studies have shown an association between fruit and vegetable intakes with the bone mineral density (BMD) in both young and elderly subjects [5], [6], [7], [8], [9], [10].

On the other hand, fruits and vegetables are also rich sources of antioxidant carotenoids, which have been shown to contribute to the body’s defense against reactive oxygen species [11], [12]. Recent animal experiments and *in vitro* studies have shown that reactive oxygen species and free radicals are involved in osteoclastogenesis, in apoptosis of osteoblasts and osteocytes, and, therefore, in bone resorption [13], [14], [15]. Furthermore, recent epidemiological studies have shown a relationship between oxidative stress and BMD or osteoporosis [16], [17], [18]. These previous findings in epidemiological and experimental studies suggest that antioxidant carotenoids may provide benefits to bone metabolism against oxidative stress. In fact, several recent epidemiological reports have shown inverse associations of antioxidant micronutrient intake or serum level with low BMD, risk of fracture, and/or risk of osteoporosis [19], [20], [21], [22], [23], [24], [25]. However, to the best of our knowledge, a thorough longitudinal cohort study about the association of serum carotenoid levels with the change of BMD and/or incidence of osteoporosis has not been conducted.

Previously, we found that the serum concentrations of β-cryptoxanthin and β-carotene were weakly but positively associated with the radial BMD in post-menopausal female subjects [26], [27]. These associations suggest that β-cryptoxanthin and β-carotene might provide benefits to bone health in post-menopausal women. However, this data consisted of cross-sectional analyses. Therefore, only limited inferences can be made regarding temporality and causation. To determine whether antioxidant carotenoids are beneficial micronutrients on bone health, further cohort study will be required.

The objective of this study was to investigate longitudinally whether the change of BMD and/or the risk of development of osteoporosis is associated with serum carotenoid concentrations. The associations of six serum carotenoid concentrations, i.e., lutein, lycopene, α-carotene, β-carotene, β-cryptoxanthin, and zeaxanthin, with BMD were evaluated longitudinally.

## Materials and Methods

### Ethics Statement

This study was carried out in accordance with the Declaration of Helsinki and approved by the ethics committee of the National Institute of Fruit Tree Science. We obtained written informed consent from all participants involved in our study.

### Study Population

This was a prospective survey involving participants in the Mikkabi cohort study conducted in the town of Mikkabi, Shizuoka Prefecture, Japan. In a baseline survey, study subjects were recruited from participants in an annual health check-up program conducted by the local government of Mikkabi in April 2005 [26]. Mikkabi is located in western Shizuoka, and about 40% of its residents work in agriculture. Fruit trees are the key industry in Mikkabi, which is an important producer of mandarin oranges in Japan. A total of 1,891 males and females were subjects for the annual health check-up program in 2005. As a result, 1,369 males and females (72.4% of total subjects), ranging in age from 30 to 70 years, had received the health check-up program. Study participants were recruited from baseline surveys, and written informed consent was obtained from 699 subjects (222 males and 477 females). The response rate was 51.1%. After four years of a baseline survey, subjects were invited to participate in a follow-up survey in April 2009. As a result, 457 subjects (146 males and 311 females) took part in the follow-up survey. The follow-up rate was 65.4%.

For the baseline survey, participants completed a self-administered questionnaire about a subject’s history of osteoporosis, hormone use, and lifestyle, including tobacco use (current smoker, ex-smoker, or non-smoker), exercise (1+ times weekly), regular alcohol intake (1+ times weekly), dietary supplement use (non-user, occasional user, current user), and dietary habits. Diet was assessed with a modified validated simple food-frequency questionnaire (FFQ) developed especially for the Japanese [28], [29]. Information about alcohol consumption and the daily intake of 18 nutrients was estimated from the monthly food intake frequencies with either standard portion size (for most types of food) or subject-specified typical portion size (for rice, bread, and alcoholic and non-alcoholic beverages) using the FFQ analysis software package for Windows (Food Frequency Questionnaire System, System Supply Co., Ltd., Kanagawa, Japan). This FFQ analysis software computes individual food and nutrient intake form FFQ data based on standard tables for food composition in Japan [30], [31]. The intakes of total energy, calcium, potassium, magnesium, and vitamins C, D, and E of each subject were used in this report.

The concentrations of six serum carotenoids at the baseline survey, lutein, lycopene, α-carotene, β-carotene, β-cryptoxanthin, and zeaxanthin, were analyzed by reverse-phase high-performance liquid chromatography (HPLC) using β-apo-8′-carotenal as an internal standard at the Laboratory of Public Health and Environmental Chemistry, Kyoto Biseibutsu Kenkyusho (Kyoto, Japan), as described previously [32].

The radial BMD at the baseline and follow-up survey was measured using dual-energy X-ray absorptiometry (DXA) of each participant’s nondominant forearm with an osteometer (model DCS-600EX-III, ALOKA Co., Ltd., Tokyo, Japan), as described previously [26]. The measurement of the radial BMD of each participant was performed by a well-trained clinical technologist of the Seirei Preventive Health Care Center (Shizuoka, Japan).

### Statistical Analysis

All subjects were categorized into three groups stratified by sex and menopausal status at the baseline survey. Serum carotenoid concentrations and intakes of vitamins C, D, and E were skewed toward the higher concentrations. These values were log_e_ (natural)-transformed to improve the normality of their distribution. The paired *t*-test was used to test differences among the baseline and follow-up survey. All variables were presented as an original scale. The data are expressed as the means (standard deviation), geometric mean (95% confidence interval), range, or percent.

The multivariate adjusted mean of the 4-year change of the radial BMD by the tertiles of the serum carotenoid concentrations at the baseline survey was calculated after adjusting for age, weight, height, years since menopause, current tobacco use, regular alcohol intake, exercise habits, supplement use, and total energy intake at the baseline using the general linear model procedure. Differences in the multivariate adjusted mean of the change of the radial BMD among each tertile of serum carotenoid concentration at the baseline were tested by Bonferroni multiple comparison. In the test for linear trends, the associations among the change of the radial BMD across three categories assigned by means of the serum carotenoid concentrations in each tertile were carried out by linear regression.

Osteoporosis was defined as a person whose T-score (which shows how a subject’s BMD compares with young adult mean) is less than 70% according to the guidelines on the management of osteoporosis by the Japan Osteoporosis Society [33]. A person whose T-score exceeds 70% and is less than 80% is defined as having osteopenia. To assess the relationship between the serum carotenoid concentrations at the baseline and the development of osteopenia and osteoporosis after four years, logistic regression analyses were performed after adjusting for age, weight, height, years since menopause, current tobacco use, regular alcohol intake, exercise habits, supplement use, and total energy intake at the baseline. Multivariable adjustment for the intakes of calcium, magnesium, potassium, and vitamins D, C, and E was further conducted as sensitivity analysis. In data analyses of the relationship between the serum carotenoid concentrations at the baseline and the development of osteoporosis, we used the data from 187 post-menopausal female subjects whose T-score at the baseline survey exceeded 70%. In a similar way, we analyzed the relationship between the serum carotenoid concentrations at the baseline and the development of osteopenia and/or osteoporosis using the data from 140 post-menopausal female subjects whose T-score at the baseline survey exceeded 80%. In the test for linear trends, the associations among the risk of osteoporosis and/or osteopenia across three categories assigned by means of the serum carotenoid concentrations in each tertile were carried out by logistic regression analysis.

We did not adjust each carotenoid concentration in the multivariate models because Pearson’s correlation analyses of serum carotenoid concentrations revealed significant positive correlations among all combinations of the six carotenoids. All statistical analyses were performed using statistical software package for Windows (SPSS ver. 12.0J, SPSS Inc., Chicago, IL, USA) on personal computers.

## Results

### Baseline Characteristics and Radial BMD Status at the Baseline and Follow-up Survey in Study Subjects

Table 1 shows the characteristics of the study subjects at the baseline survey stratified by sex and menopausal status at the baseline. Among pre-menopausal female subjects at the baseline survey, 35 subjects had reached menopause.

**Table 1:** Caracteristics of the study subject at baseline survey^a.^

	Male		Pre-menopausal female		Post-menopausal female	
N	146		99		212	
Age (y)	57.1	(8.9)	44.8	(5.3)	60.5	(5.8)
Body height (cm)	165.8	(6.5)	155.4	(4.9)	151.9	(5.7)
Body weight (kg)	64.9	(8.7)	54.9	(9.7)	50.9	(7.4)
Body mass index (kg/m^2^)	23.6	(2.7)	22.7	(3.9)	22.0	(2.8)
Total energy intake (kcal/day)						
Including ethanol	2157.4	(473.8)	1913.7	(437.5)	1940.5	(443.2)
Excluding ethanol	2017.7	(452.2)	1898.2	(441.6)	1928.0	(443.1)
Calcium intake (mg/day)	521.3	(219.3)	584.6	(190.8)	642.1	(232.2)
Potassium intake (mg/day)	2481.0	(824.5)	2512.2	(774.2)	2871.1	(860.7)
Magnesium intake (mg/day)	270.3	(72.5)	244.5	(66.1)	278.4	(72.6)
Vitamin C intake (mg/day)^b^	126.3	(115.3–138.4)	115.4	(104.7–127.2)	170.6	(161.0–180.8)
Vitamin D intake (µg/day)^b^	4.9	(4.4–5.5)	5.1	(4.5–5.7)	6.3	(5.8–6.8)
Vitamin E intake (mg/day)^b^	7.4	(7.0–7.9)	7.6	(7.2–8.1)	8.0	(7.7–8.3)
Serum carotenoid concentrations (µmol/L)^b^						
Lutein	0.45	(0.43–0.47)	0.47	(0.44–0.50)	0.56	(0.53–0.58)
Lycopene	0.31	(0.28–0.33)	0.46	(0.42–0.50)	0.38	(0.35–0.40)
α-Carotene	0.13	(0.12–0.14)	0.20	(0.18–0.22)	0.22	(0.20–0.24)
β-Carotene	0.58	(0.53–0.65)	0.90	(0.81–1.01)	1.16	(1.09–1.24)
β-Cryptoxanthn	1.26	(1.09–1.45)	1.05	(0.91–1.22)	1.80	(1.64–1.98)
Zeaxanthin	0.19	(0.18–0.20)	0.20	(0.19–0.21)	0.21	(0.20–0.21)
Current tobacco use (%)	25.3		2.0		1.9	
Exercise habits (%)^c^	21.2		14.1		17.9	
Regular alcohol intake (%)^c^	58.9		16.2		13.2	
Current supplement use (%)	3.4		14.1		9.0	

a Data are mean (standard deviation), geometric mean (95% confidence interval), range, or percent.

b These variables were represented as original scale after analysis by log (natural) transformed values.

c > = 1 times/wk.

Table 2 shows the radial BMD at the baseline survey and follow-up surveys stratified by sex and menopausal status at the baseline. The radial BMD at the follow-up survey in male and pre- and post-menopausal female subjects was significantly lower than that in each group at the baseline survey. Osteopenia, as newly diagnosed for male and pre-menopausal female subjects in our study, was identified in only two males and one female. None of the males or pre-menopausal females developed osteoporosis. On the other hand, in post-menopausal female subjects, two subjects in the normal group at the baseline and fifteen subjects in the osteopenia group at the baseline developed osteoporosis after four years. Furthermore, twenty-five subjects in the normal group at the baseline developed osteopenia. In our survey, two subjects identified as having osteoporosis at the baseline were identified as having osteopenia during the follow-up survey. Three subjects with osteopenia were diagnosed as normal during the follow-up survey. The radial BMD in these five subjects seemed to increase over the four-year follow-up.

**Table 2:** Bone mineral density and T-score at baseline and follow-up survey^a.^

	Male		Pre-menopausal female^b^		Post-menopausal female	
n	146		99		212	
Baseline survey in 2005						
Bone mineral density (g/cm^2^)	0.770	(0.064)	0.680	(0.056)	0.554	(0.085)
Range		0.593–0.906		0.521–0.817		0.380–0.820
T-Score (%)	99.8	(8.2)	105.2	(8.7)	85.8	(13.2)
Normal [n, (%)]	144	(98.6)	99	(100.0)	140	(66.0)
Osteopenia [n, (%)]	2	(1.4)	0	(0.0)	47	(22.2)
Osteoporosis [n, (%)]	0	(0.0)	0	(0.0)	25	(11.8)
Follow-up survey in 2009						
Bone mineral density (g/cm^2^)	0.765	(0.069)^c^	0.669	(0.062)^c^	0.527	(0.084)^c^
Range		0.565–0.913		0.482–0.803		0.330–0.725
T-Score (%)	99.1	(8.8)^c^	103.6	(9.6)^c^	81.7	(13.0)^c^
Normal [n, (%)]	142	(97.3)	98	(99.0)	116	(54.7)
Osteopenia [n, (%)]	4	(2.7)	1	(1.0)	56	(26.4)
Osteoporosis [n, (%)]	0	(0.0)	0	(0.0)	40	(18.9)

a Data are mean (standard deviation), range, or percent.

b Newly thirty five female subjects reached menopause four years later.

c P<0.001 vs baseline by paired *t*-test.

In our follow-up survey, the baseline radial BMD in male and pre-menopausal female subjects was no different among fallout and follow-up subjects (data not shown). On the other hand, in post-menopausal female subjects, the mean of the baseline radial BMD in fallout subjects was significantly higher than that in subjects who participated in the follow-up survey (P = 0.019).

### Multivariate Adjusted Means of the Change of Radial BMD for Four Years by Stratified Tertiles of each Serum Carotenoid Concentration

**Figure 1:**
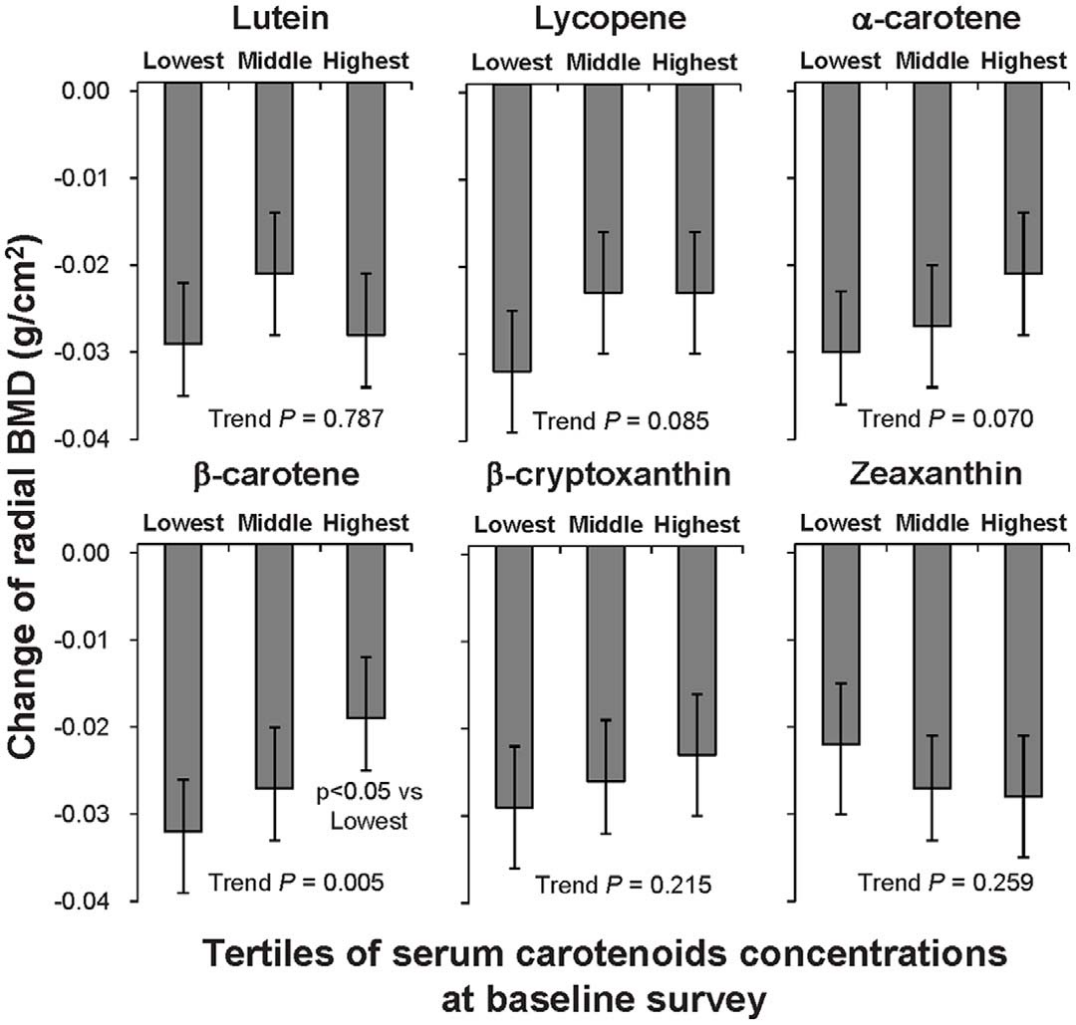
Change of radial bone mineral density by tertiles of serum carotenoids in post-menopausal female subjects. Multivariate-adjusted means of radial bone mineral density were calculated after adjusting for age, weight, height, years since menopause, current tobacco use, regular alcohol intake, exercise habits, supplement use, and total energy intake. *P*-values over the quartiles of serum carotenoids were assessed with a test for linear trends using linear regression.

Figure 1 shows the multivariate-adjusted means of the change of the radial BMD for four years by the tertiles of the baseline serum carotenoid concentrations among post-menopausal female subjects. The change of the radial BMD in the highest tertile of serum β-carotene was significantly lower than that in the lowest one. This significant difference was also observed after further adjusting for intakes of calcium, magnesium, potassium, and vitamins D, C, and E (P for linear tend: 0.002). In contrast, in male and pre-menopausal female subjects, no significant associations were observed in any of the six serum carotenoids (data not shown).

Although newly thirty-five female subjects reached menopause four years later, significant associations between the changes of the radial BMD and baseline serum carotenoid concentration were not observed in either pre-menopausal female subjects or newly post-menopausal female subjects who were pre-menopausal at the baseline (data not shown).

### Risk of Osteoporosis According to Tertiles of Baseline Serum Carotenoid Concentrations

The odds ratios of osteoporosis associated with the tertiles of six serum carotenoid concentrations at the baseline survey after adjusting for confounding factors are shown in Table 3. After excluding subjects whose T-scores were under 70% at the baseline survey, the odds ratios of osteoporosis in the middle (T2) and highest (T3) groups against the lowest tertile (T1) used for the reference group were calculated. After adjusting for age, weight, height, years since menopause, current tobacco use, regular alcohol intake, exercise habits, supplement use, and total energy intake, a significant lower odds ratio for osteoporosis among post-menopausal female subjects was observed in the highest group (T3) of serum β-cryptoxanthin. This inverse association between baseline serum β-cryptoxanthin and the development of osteoporosis was also observed after further adjusting for the intakes of calcium, magnesium, potassium, and vitamins C, D, and E. On the other hand, significantly lower odds ratios were not observed in other carotenoids. In this study population, although four post-menopausal female subjects were currently using female hormones, such as estrogen, the associations of the radial BMD with serum carotenoid concentrations did not change after excluding these four subjects.

**Table 3:** Odds ratios (and 95% confidence intervals) of tertiles of baseline serum carotenoid concentration on osteoporosis in post-menopausal Japanese female subjects.

Serum carotenoids		N	Mean and range of serum carotenoid (µmol/L)		Case	Model 1			Model 2		
						OR	95% CI	*P* for trend	OR	95% CI	*P* for trend
Lutein	Lowest (T1)	60	0.38	(0.23–0.47)	6	1.00			1.00		
	Middle (T2)	66	0.56	(0.49–0.63)	6	0.69	(0.19–2.51)		0.47	(0.10–2.13)	
	Highest (T3)	61	0.78	(0.65–2.11)	5	0.53	(0.14–2.05)	0.356	0.54	(0.11–2.60)	0.433
Lycopene	Lowest (T1)	59	0.23	(0.07–0.32)	7	1.00			1.00		
	Middle (T2)	61	0.38	(0.34–0.45)	4	0.71	(0.17–2.97)		0.57	(0.11–2.81)	
	Highest (T3)	67	0.62	(0.47–1.06)	6	0.91	(0.25–3.37)	0.890	0.58	(0.14–2.51)	0.481
α-Carotene	Lowest (T1)	67	0.13	(0.07–0.17)	7	1.00			1.00		
	Middle (T2)	56	0.21	(0.19–0.22)	5	0.80	(0.22–2.94)		0.64	(0.13–3.09)	
	Highest (T3)	64	0.38	(0.24–2.74)	5	0.76	(0.20–2.94)	0.694	0.71	(0.14–3.68)	0.733
β-Carotene	Lowest (T1)	61	0.69	(0.35–0.91)	9	1.00			1.00		
	Middle (T2)	64	1.17	(0.95–1.42)	4	0.32	(0.08–1.28)		0.26	(0.05–1.24)	
	Highest (T3)	62	1.91	(1.43–3.37)	4	0.30	(0.08–1.13)	0.071	0.24	(0.05–1.21)	0.082
β-Cryptoxanthin	Lowest (T1)	62	0.81	(0.24–1.41)	9	1.00			1.00		
	Middle (T2)	63	1.87	(1.43–2.39)	7	0.83	(0.24–2.90)		0.63	(0.16–2.42)	
	Highest (T3)	62	3.60	(2.41–10.53)	1	0.08	(0.01–0.75)	0.021	0.07	(0.01–0.88)	0.034
Zeaxanthin	Lowest (T1)	56	0.15	(0.09–0.18)	8	1.00			1.00		
	Middle (T2)	64	0.20	(0.19–0.21)	5	0.50	(0.14–1.84)		0.50	(0.12–2.04)	
	Highest (T3)	67	0.26	(0.23–0.37)	4	0.36	(0.09–1.38)	0.130	0.35	(0.08–1.52)	0.149

Model 1: Age, weight, height, years since menopause, current tobacco use, regular alcohol intake, exercise habits, supplement use, and total energy intake were adjusted.

Model 2: Intakes of calcium, magnesium, potassium, and vitamins D, C, and E were further adjusted.

### Risk of Osteopenia and/or Osteoporosis According to Tertiles of Baseline Serum Carotenoid Concentrations

The odds ratios of osteopenia and/or osteoporosis associated with the tertiles of six serum carotenoid concentrations at the baseline survey after adjusting for confounding factors are shown in Table 4. After excluding subjects whose T-scores were under 80% at the baseline survey, the odds ratios of osteopenia and/or osteoporosis in the middle (T2) and highest (T3) groups against the lowest tertile (T1) used for the reference group were calculated. After adjusting for age, weight, height, years since menopause, current tobacco use, regular alcohol intake, exercise habits, supplement use, and total energy intake, significant lower odds ratios for osteopenia and/or osteoporosis among post-menopausal female subjects were observed in the middle (T2) and highest group (T3) with high serum β-cryptoxanthin. These significant lower odds ratios were also observed after multivariate adjustment. On the other hand, significant lower risks for osteopenia and/or osteoporosis were not observed in other carotenoids.

**Table 4:** Odds ratios (and 95% confidence intervals) of tertiles of baseline serum carotenoid concentration on osteopenia and/or osteoprosis in post-menopausal Japanese female subjects.

Serum carotenoids		n	Mean and range of serum carotenoid (µmol/L)		Case	Model 1			Model 2		
						OR	95% CI	*P* for trend	OR	95% CI	*P* for trend
Lutein	Lowest (T1)	47	0.39	(0.28–0.47)	8	1.00			1.00		
	Middle (T2)	45	0.55	(0.49–0.62)	9	1.48	(0.46–4.68)		1.50	(0.45–5.01)	
	Highest (T3)	48	0.74	(0.63–1.14)	10	1.29	(0.41–4.08)	0.676	1.62	(0.47–5.56)	0.445
Lycopene	Lowest (T1)	48	0.24	(0.07–0.34)	7	1.00			1.00		
	Middle (T2)	46	0.40	(0.35–0.46)	10	1.61	(0.52–5.00)		2.04	(0.61–6.83)	
	Highest (T3)	46	0.66	(0.50–1.06)	10	1.54	(0.46–5.13)	0.481	1.57	(0.42–5.90)	0.480
α-Carotene	Lowest (T1)	46	0.13	(0.07–0.17)	8	1.00			1.00		
	Middle (T2)	46	0.21	(0.19–0.24)	9	1.18	(0.39–3.54)		1.55	(0.46–5.18)	
	Highest (T3)	48	0.39	(0.26–2.75)	10	0.89	(0.29–2.73)	0.806	0.97	(0.28–3.31)	0.889
β-Carotene	Lowest (T1)	47	0.70	(0.35–0.97)	9	1.00			1.00		
	Middle (T2)	47	1.23	(0.99–1.45)	10	0.78	(0.26–2.36)		0.87	(0.28–2.73)	
	Highest (T3)	46	1.94	(1.50–3.36)	8	0.56	(0.18–1.76)	0.324	0.58	(0.18–1.91)	0.378
β-Cryptoxanthin	Lowest (T1)	46	0.85	(0.24–1.45)	14	1.00			1.00		
	Middle (T2)	47	1.95	(1.50–2.44)	6	0.22	(0.07–0.76)		0.18	(0.05–0.67)	
	Highest (T3)	47	3.78	(2.47–10.51)	7	0.29	(0.09–0.96)	0.037	0.28	(0.08–0.99)	0.035
Zeaxanthin	Lowest (T1)	41	0.15	(0.11–0.18)	7	1.00			1.00		
	Middle (T2)	50	0.20	(0.19–0.21)	11	1.40	(0.44–4.42)		1.52	(0.46–4.99)	
	Highest (T3)	49	0.26	(0.23–0.37)	9	1.28	(0.40–4.14)	0.697	1.42	(0.41–4.91)	0.593

Model 1: Age, weight, height, years since menopause, current tobacco use, regular alcohol intake, exercise habits, supplement use, and total energy intake were adjusted.

Model 2: Intakes of calcium, magnesium, potassium, and vitamins D, C, and E were further adjusted.

### Baseline Serum Carotenoid Concentrations among Continuously Normal Subjects, Newly Diagnosed Osteopenia, and Newly Diagnosed Osteoporosis

Table 5 shows the baseline serum carotenoid concentrations of post-menopausal female subjects stratified by continuously normal subjects, newly diagnosed osteopenia, and newly diagnosed osteoporosis defined by T-score levels at the follow-up survey. In our survey, in post-menopausal female subjects, newly seventeen subjects developed osteoporosis and newly twenty-five subjects developed osteopenia over the course of four years. A total of 113 subjects were diagnosed as continuously normal at the baseline and throughout the follow-up surveys. After multivariate adjustment, the baseline serum β-cryptoxanthin of subjects who were newly diagnosed osteoporosis was significantly lower than that of continuously normal subjects. Furthermore, the baseline serum β-carotene also tended to be low; however, it was not statistically significant.

**Table 5:** Baseline serum carotenoid concentrations of post-menopausal Japanese female subjects stratified by T-Score levels in 2009.

	Mean and range of serum carotenoid concentrations (µmol/L)						
	Continuously normal		Newly diagnosed osteopenia		Newly diagnosed osteoporosis		P for trend
N	113		25		17		
Lutein	0.54	(0.51–0.57)	0.56	(0.50–0.63)	0.52	(0.44–0.61)	0.660
Lycopene	0.39	(0.36–0.43)	0.41	(0.34–0.49)	0.38	(0.30–0.48)	0.969
α-Carotene	0.23	(0.20–0.25)	0.21	(0.17–0.27)	0.21	(0.16–0.28)	0.370
β-Carotene	1.22	(1.13–1.33)	1.06	(0.89–1.27)	0.97	(0.78–1.21)	0.025
β-Cryptoxanthin	1.94	(1.72–2.19)	1.59	(1.22–2.07)	1.16	(0.85–1.58)*	<0.001
Zeaxanthin	0.20	(0.19–0.21)	0.21	(0.19–0.23)	0.18	(0.16–0.21)	0.388

Age, weight, height, years since menopause, current tobacco use, regular alcohol intake, exercise habits, supplement use, and total energy intake were adjusted.

* p<0.01 vs Normal group by Bonferroni multiple comparison test.

## Discussion

The objective of this study was to investigate longitudinally whether the change of BMD is associated with serum carotenoid concentration. The results indicated that higher serum β-carotene at the baseline in post-menopausal female subjects was significantly associated with less bone loss of the radius four years later. Furthermore, high serum β-cryptoxanthin was associated with a lower risk of osteoporosis and/or osteopenia. In addition, our retrospective analysis revealed that subjects who developed osteoporosis and/or osteopenia during the survey period had significantly lower serum concentrations of β-cryptoxanthin and β-carotene at the baseline compared to the continuously normal group. In other words, subjects who had had higher serum concentrations of β-cryptoxanthin and β-carotene at the baseline were less likely to develop osteoporosis and/or osteopenia in later years. Numerous antioxidant vitamins and carotenoids are contained in fruits and vegetables, and several recent epidemiological reports have shown inverse associations of antioxidant vitamin and carotenoid intake or serum level with low BMD, risk of fracture, and/or risk of osteoporosis [19], [20], [21], [22], [23], [24], [25]. Previously, we found that the serum concentrations of β-cryptoxanthin and β-carotene were weakly but positively associated with the radial BMD in post-menopausal female subjects from the cross-sectional analyses [26], [27]. However, to the best of our knowledge, thorough longitudinal cohort studies about the association of bone loss with serum carotenoid concentration have not been conducted. This is the first longitudinal cohort study to examine the association of the serum carotenoid concentration with the change of BMD. Our findings further support the hypothesis that high intakes of fruit and vegetables rich in antioxidant carotenoids, especially β-cryptoxanthin and β-carotene, might provide benefits to bone health in post-menopausal female subjects.

Recent studies have also implicated the possible involvement of oxidative stress in BMD loss and increased risk of fractures. In fact, experimental evidence shows that smokers have increased risk of fractures [18], [19], that osteoporosis patients have lower concentrations of serum vitamin C and vitamin E and exhibit elevated serum oxidative stress marker levels [16], [17], [19], [20], and that NF-κB proteins that play an important role in bone resorption become activated when exposed to oxidative stress [34], [35], [36], [37]. Although all types of carotenoids are known to have potent antioxidative properties, no association was found among carotenoids other than β-cryptoxanthin and β-carotene and BMD in our study. This finding suggests that mechanisms other than antioxidant activity might be involved in the effect of carotenoids on bone metabolism. Further research is needed.

Our observation is consistent with experimental results previously reported. Recently, Yamaguchi et al. reported the beneficial effects of β-cryptoxanthin on bone metabolism in *in vitro* and *in vivo* studies [38], [39], [40]. They found [38] that β-cryptoxanthin enhanced the calcium content and alkaline phosphatase activity in the femoral-diaphyseal and femoral-metaphyseal tissues of young rats at physiological low concentrations *in vitro*, while lycopene and lutein had no effects at the same dose. Furthermore, they found [39] a stimulatory effect on bone formation and an inhibitory effect on bone resorption in a tissue culture. In an *in vivo* study, they found [40] that the oral administration of β-cryptoxanthin caused a significant increase in the calcium content and alkaline phosphatase activity in the femoral-diaphyseal and femoral-metaphyseal tissues. These previous results support our findings that β-cryptoxanthin may have a direct stimulatory effect on bone formation and an inhibitory effect on bone resorption. The development of osteoporosis may be reduced by a dietary intake of β-cryptoxanthin.

Several epidemiological research teams are currently examining longitudinally the possible association of carotenoids with osteoporosis, changes in BMD, or risks of fractures. In the past, most epidemiological studies on the association between carotenoids and bone health have been carried out using either cross-sectional, case-control, and/or nested case-control designs to examine carotenoid intake and how it might affect BMD or bone health. A few studies assessing this association using the serum carotenoid levels were based on cross-sectional or case-control studies. According to these studies, lycopene, β-carotene, and β-cryptoxanthin have all been reported to be associated with bone health [21], [22], [23], [24].

It is important to consider that dietary habits in populations vary across studies. Thus, studies conducted in Europe or the U.S., where lycopene intake is relatively high, tend to implicate lycopene as the type of carotenoid affecting bone metabolism. On the other hand, a few studies [22], [23] targeting Italian and/or American populations have reported on the efficacy of β-cryptoxanthin. In recent years, the Framingham research group [24] published results from a longitudinal analysis of the association between carotenoid intake and BMD. This group analyzed the effects of carotenoid intake and associated changes in BMD over four years and concluded that a high intake of lycopene has inhibitory effects on BMD loss. Nonetheless, they found no association between β-cryptoxanthin and bone health.

In general, there is little available epidemiological research on the association between carotenoids and bone health. No studies have, in fact, been conducted to date to longitudinally analyze the association between serum carotenoid levels and bone health. In this respect, ours is the first study including a longitudinal analysis of the association between serum carotenoid levels and BMD and demonstrating the possible inhibitory effects of β-cryptoxanthin and β-carotene on BMD loss. In general, when the carotenoid intake of each subject is estimated on the basis of data collected from a diet survey, the estimated intake levels may not necessarily reflect the actual amount of carotenoid ingested or absorbed into the body. On the other hand, data on serum carotenoid levels are a relatively accurate measure of the actual amount of carotenoids present in the body, which therefore gives a more detailed account of the association between carotenoid levels and BMD.

In contrast to findings collected in Europe and the U.S., no significant association has been observed between lycopene and bone health in the subjects who participated in our study. This may be partly due to the fact that the groups of subjects in the Mikkabi study have much lower dietary intake and serum levels of lycopene than individuals living in other parts of the world. Similarly, although a higher serum concentration of α-carotene has contributed to some extent to a decreasing rate of BMD loss, the association has not been found to be significant. A more extended follow-up may reveal a significant association. On the other hand, our analyses failed to show any association of lutein and zeaxanthin with BMD. In a previous study, lutein was found to be consumed in the highest amount by the subjects in the Mikkabi study, and the serum concentration of lutein was also found to be higher than those of α-carotene or lycopene [41]. However, no association was found between lutein and bone health. This finding suggests that none of these carotenoids is involved in the maintenance of bone health.

This study had some limitations. First, we could not evaluate the association of blood levels of vitamins C and E with the radial BMD. Some studies have shown an association of antioxidant vitamins with the risk of hip fracture in current smokers and aged osteoporotic women [19], [20]. It would be necessary to measure blood levels of vitamins C and E in order to examine the associations of these antioxidant vitamin concentrations with the radial BMD. Second, in this report, we evaluated the radial BMD at 1/3 of the forearm length measured from the styloid process on the ulna. Therefore, an analysis of the association of serum carotenoids with BMD in cancellous bone, such as the femoral neck or lumbar spine, will be required. Last, in our study, sample size in post-menopausal female subjects was not so large and it had less statistical power. Further study in large scale will be required.

Our findings lead us to conclude that β-cryptoxanthin and β-carotene are probably the two types of carotenoids that are involved in the prevention of BMD loss in post-menopausal Japanese women. However, further evidence from epidemiological research is needed before a definitive conclusion on this issue can be drawn.
